# Dietary Intervention, When Not Associated With Exercise, Upregulates Irisin/FNDC5 While Reducing Visceral Adiposity Markers in Obese Rats

**DOI:** 10.3389/fphys.2021.564963

**Published:** 2021-08-13

**Authors:** Vanessa de Oliveira Furino, João Manoel Alves, Diego Adorna Marine, Marcela Sene-Fiorese, Carla Nascimento dos Santos Rodrigues, Cristina Arrais-Lima, Stela Márcia Mattiello, Cynthia Aparecida de Castro, Ricardo Carneiro Borra, Marina Campos Rocha, Iran Malavazi, Ana Cláudia Garcia de Oliveira Duarte

**Affiliations:** ^1^Department of Physical Education and Human Motricity – DEFMH, Biological and Health Sciences Center – CCBS, Federal University of São Carlos – UFSCar, São Carlos, Brazil; ^2^Department of Physiotherapy – DFisio-Biological and Health Sciences Center – CCBS, Federal University of São Carlos – UFSCar, São Carlos, Brazil; ^3^Department of Morphology and Pathology-Biological and Health Sciences Center – CCBS, Federal University of São Carlos – UFSCar, São Carlos, Brazil; ^4^Department of Genetics and Evolution-Biological and Health Sciences Center – CCBS, Federal University of São Carlos – UFSCar, São Carlos, Brazil

**Keywords:** body composition, endurance training, visceral adipose tissue, high-fat diet, obesity, irisin/FNDC5

## Abstract

Obesity is an epidemic disease and the expansion of adipose tissue, especially visceral fat, promotes the secretion of factors that lead to comorbidities such as diabetes and cardiovascular diseases. Thus, diet and exercise have been proposed as an intervention to reverse these complications. An adipocytokine, known as irisin, mediates the beneficial effects of exercise. It has been proposed as a therapeutic potential in controlling obesity. In view of the above, this paper attempts to determine the modulation of irisin, visceral adiposity and biochemical markers in response to dietary intervention and aerobic exercise. To do this, 52 diet-induced obese male *Wistar* rats were divided into the following four groups: high-fat diet and exercise (HFD-Ex); HFD-Sedentary (HFD-Sed); chow-diet and exercise (CD-Exercise); and CD-Sed. The exercise-trained group performed a treadmill protocol for 60 min/day, 3 days/week for 8 weeks. Body mass (BM), body fat (BF), fat mass (FM), and fat-free mass (FFM) were analyzed. Mesenteric (MES), epididymal (EPI), and retroperitoneal (RET) adipose tissue was collected and histological analysis was performed. Biochemical irisin, triglycerides, glucose, insulin and inflammatory markers were determined and, FNDC5 protein expression was analyzed. In this study, the diet was the most important factor in reducing visceral adiposity in the short and long term. Exercise was an important factor in preserving muscle mass and reducing visceral depots after a long term. Moreover, the combination of diet and exercise can enhance these effects. Diet and exercise exclusively were the factors capable of increasing the values of irisin/FNDC5, however it did not bring cumulative effects of both interventions. Prescriptions to enhance the obesity treatments should involve reducing visceral adiposity by reducing the fat content in the diet associated with aerobic exercise.

## Introduction

Obesity is the result of an increase in the intake of a high-fat and high-carbohydrate diet associated with low levels of physical activity (Romieu et al., 2017). A chronic state of positive energy balance, derived from this condition, promotes the unhealthy expansion of visceral and subcutaneous adipocytes, inducing a remodeling of adipose tissue (Bray et al., 2017; Schoettl et al., 2018). A growing body of evidence shows that obesity-related comorbidities are influenced by adipocyte distribution and more specifically visceral fat. Progressive enlargement of visceral adipose tissue (VAT) causes alterations to mitochondrial oxidative function, increases lipolytic activity induced by catecholamine, and the secretion of pro-inflammatory cytokines leading to chronic inflammation and subsequent dysfunction bioenergetics and structural changes in adipocytes (Wajchenberg, 2000; Kusminski et al., 2016). This dysfunction is associated with an array of metabolic complications, such as type 2 diabetes and cardiovascular disease (Bray et al., 2017; Schoettl et al., 2018).

Due to the high remodeling capacity of white adipose tissue (WAT) and endocrine functions, preserving healthy WAT function and decreasing adiposity, especially visceral fat, has been considered an attractive approach for the treatment or prevention of metabolic disorders related to obesity (Verheggen et al., 2016; Kahn et al., 2019). Thus, exercise and diet have been proposed as non-pharmacological strategies for VAT management (Verheggen et al., 2016). Indeed, diet plays an important role in weight reduction, recent data has shown that exercise is a predominant factor in the regulation of VAT when compared to a hypocaloric diet (Verheggen et al., 2016). Visceral fat reduction is associated with a decrease in body mass (BM) and fat mass (FM), improvement of glucose homeostasis, lipid profile, and reduction of an inflammatory state (Verheggen et al., 2016; Chait and den Hartigh, 2020). The mechanism involved in this regulation is not completely understood, but it can be suggested that the muscle considering high energy demand, secretes factors that stimulate the thermogenesis of WAT (Rodríguez et al., 2017).

Among the secreted factors, irisin is a novel exercise-induced adipomyokine cleaved of FNDC5 (fibronectin type III domain-containing protein 5), a transmembrane protein expressed in muscle (Boström et al., 2012). It is believed that after secretion into the circulation, soluble irisin binds to a recently identified irisin receptor, integrin αV/β5, that induces a thermogenic program (Kim et al., 2018). The transcription of thermogenic genes regulates the mitochondrial activity and increases energy expenditure, transdifferentiating white adipocytes to brown-like phenotype adipocytes (Cheng et al., 2021). Besides, studies have also shown that irisin facilitates glucose uptake by skeletal muscles, increases insulin sensitivity of tissues, stimulates mitochondrial biogenesis and oxidative metabolism, improving the metabolic profile (Boström et al., 2012; Rodríguez et al., 2017) and attenuates the expression of obesity-related inflammatory markers (Lu and Li, 2020; Luo et al., 2020). Since then, several studies show that short-term aerobic exercises (Anastasilakis et al., 2014; Aydin et al., 2014) and long-term exercises (Boström et al., 2012; Kim et al., 2016) upregulate FNDC5 and irisin levels in humans and animals. Although the main source of expression is exercise/muscle, VAT and subcutaneous adipose tissue (SAT) also secretes irisin in a reduced amount against different nutritional states (Frühbeck et al., 2020). For instance, obesity seems to positively regulate the concentrations of irisin in adipocytes and muscles to respond to an uncommon metabolic condition (Park et al., 2013; Crujeiras et al., 2014; Pardo et al., 2014; Sahin-Efe et al., 2018). However, a negative correlation has already been observed between circulating levels of irisin, BMI, and the amount of adipose tissue (Bonfante et al., 2017; Grygiel-Górniak and Puszczewicz, 2017) and reductions in plasma concentrations in patients with morbid obesity (Frühbeck et al., 2020).

Finally, in addition to the aforementioned factors that interfere with the circulation of irisin, among the adipose tissue compartments, there are differences in the protein secretion of each depot. There is strong evidence indicating irisin is upregulated in VAT, but not in SAT in human adipose tissue (Frühbeck et al., 2020), justified by the specific characteristics of these compartments (Roca-Rivada et al., 2013). It is even suggested that the thermogenic capacity of SAT is conferred by irisin (Arhire et al., 2019).

Many studies have compared irisin/FNDC5 in VAT and SAT, but very little is currently known about the responses among the different visceral compartments. The VAT is a heterogeneous tissue and the epididymal (EPI), mesenteric (MES), and retroperitoneal (RET) depots regulate energy metabolism uniquely (Wronska and Kmiec, 2012; Schoettl et al., 2018). For this reason, we consider that irisin is modulated in a particular way with each depot and this difference results in specific metabolic adaptations during the obesity process. Therefore, understanding the responses of each depot, as well as specific factors secreted by each tissue, is relevant.

Part of the low efficiency in the management of obesity is due to the knowledge gap that still persists in factors that act in the regulation of BM and FM. Thus, additional studies are needed to explore the factors secreted by the adipose and muscular tissue that promote corrective and physiological actions in the obesity process and in response to the strategies for the treatment of obesity. In view of the above, this paper attempts to determine the modulation of irisin and inflammatory markers, visceral adiposity parameters and depots in response to dietary intervention and moderate-intensity exercise. We hypothesize that exercise training exclusively increases the secretion of irisin and improves dyslipidemia, improves glucose homeostasis, reduces inflammatory markers and the effects can be intensified from a shift from a high-fat diet (HFD) to a chow diet (CD).

## Materials and Methods

### First Intervention (Diet-Induced Obesity)

**FIGURE 1:**
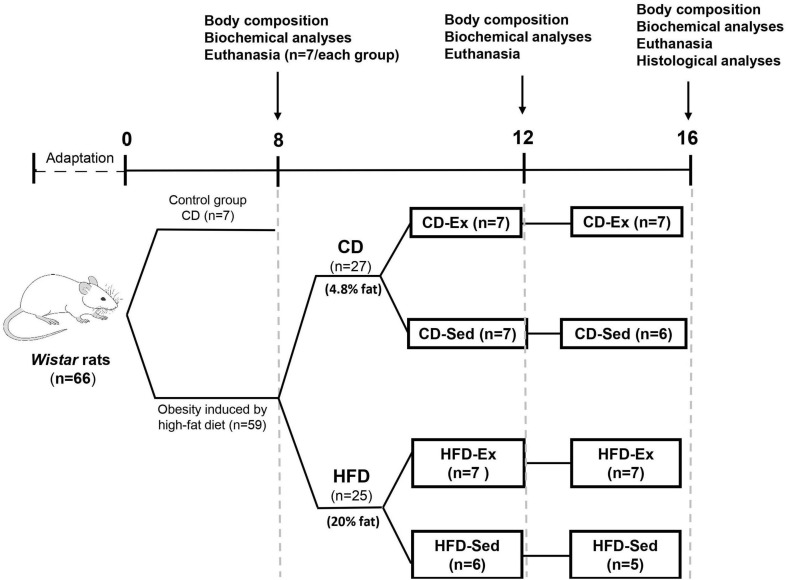
Schematic representation of the study design. HFD: high-fat diet; CD: chow diet; HFD-Ex: high-fat diet and exercise; HFD-Sed: sedentary high-fat diet; CD-Ex: chow diet and exercise; CD-Sed: sedentary chow diet.

Experimental protocols were approved by the Ethics Committee on the Use of Animals (no.7631210617) at the Federal University of São Carlos (UFSCar). As shown in Figure 1, the experimental protocol lasted 16 weeks. Adult male *Wistar* rats (*n* = 66; ≅ 300 g) were housed (*n* = 3–4/cage) in a temperature-controlled environment (23 ± 1°C) and humidity (50–60%) on a reversed 12/12 h light/dark cycle (lights on at 6 pm) with food and water *ad libitum.*

After adaptation, rats were randomly divided to be fed one of the following diets over 8 weeks: CD (*n* = 7) or HFD (*n* = 59). At the end of the first intervention (Figure 1), 7 animals of the CD group and 7 animals of the HFD group were euthanized to assess the efficiency of the HFD in inducing obesity and for further analysis. The remaining 52 animals in the HFD group proceeded to the subsequent intervention (training and dietary intervention).

### Second Intervention (Training and Dietary Intervention)

After 8 weeks on HFD, 52 animals were randomly divided into four groups (Figure 1): high-fat diet and exercise (HFD-Ex, *n* = 14), sedentary high-fat diet (HFD-Sed, *n* = 11), and two groups that switched to CD; chow diet and exercise (CD-Ex, *n* = 14) and sedentary chow diet (CD-Sed, *n* = 13) for 8 more weeks. After 4 and 8 weeks during the second intervention, 5–7 animals from each group were euthanized after a 12-h fast by guillotine decapitation between 8 am and 12 pm. The trained animals were sacrificed 48 h after the last physical activity to assess body composition and biochemical parameters.

### Diets

The CD (in pellet form) was provided by Agromix (Jaboticabal, SP, Brazil) that contained (100 g) 23% protein, 39% carbohydrates, 4.8% total fat, and 6% fiber. The palatable HFD consists of standard chow diet, peanuts, milk chocolate, and sweet biscuit at a proportion of 3:2:2:1. All components were powdered and mixed to form pellets (Estadella et al., 2004). The diet contains 18% protein, 20% fat, 33% carbohydrate, and 3% fiber (100 g). The caloric densities were 4.66 kcal/g for the palatable high fat diet and 3.85 kcal/g for the chow diet (IKA 5000, CBO, Valinhos, Brazil). The complete description of the macronutrients and vitamins was previously described (Costa et al., 2021).

### Training Protocol

#### Exercise Maximum Capacity Assessment

All animals were adapted to the treadmill (10–15 min/day; 6 to 10 m/min) for 5 days before beginning the exercise training protocol. At the end of the adaptation and in order to determine the running speed for the training protocol, the exercise intensity was estimated by the total distance covered and the maximum speed obtained in the maximum test protocol (Brooks and White, 2018). The progressive effort test on the treadmill comprised increments at a speed of 2 m/min every 2 min until the maximum speed was obtained (Souza et al., 2018). The exhaustion time (in min) and the maximum speed (m/min) were determined as 100% of the exercise capacity and used to determine the intensity of the training sessions (Table 1). During the procedure, an electric shock was not used as a form of stimulation.

**TABLE 1:** Maximal incremental exercise testing.

	Baseline		Week 4		Week 8	
	HFD-Ex	CD-Ex	HFD-Ex	CD-Ex	HFD-Ex	CD-Ex
V_m__a__x_ (m/min)	25.7 ± 0.6^a^	21.5 ± 0.3	30.6 ± 0.7^b^	30.1 ± 1.0^b^	33.1 ± 0.6^b^	34.8 ± 1.1^b,c^
Δ*t* (min)	19.6 ± 0.8^a^	15.6 ± 0.6	26.9 ± 0.5^a,b^	26.0 ± 1.1^b^	29.3 ± 0.6^b^	30.7 ± 0.9^b,c^
Δ*s* (m)	508.0 ± 30.0^a^	337.7 ± 16.1	825.2 ± 31.2^b^	798.9 ± 64.0^b^	972.6 ± 36.6^b^	1076.0 ± 65.8^b,c^

HFD-Ex: high-fat diet and exercise; CD-Ex: chow diet and exercise; *V*_*max*_: maximum speed; Δ*t*: elapsed time; Δ*s*: distance covered. The results are presented as means ± SEM. *p < 0.05 vs. CD.

*^*a*^vs. CD-Ex in the same week.*

*^*b*^vs. baseline in the same group.*

*^*c*^vs. week 4 in the same group.*

#### Treadmill Training Protocol

The training protocol consisted of running sessions on a treadmill adapted for rats, containing six individual lanes separated by bays made of acrylic, always between 8 am and 12 pm corresponding to the dark cycle of the animals. The aerobic training protocol had a frequency of 3 weekly sessions for 8 weeks, lasting 60 min per session, at an intensity of 50–80% of the maximum speed obtained in the progressive effort test, with a slope of 0%. Each training session was divided into three parts, 10 min for warm-up (50–60% of *V*_max_), 40 min for the main part (65–80% of *V*_max_) and 10 min of gradual speed reduction (50% of *V*_max_).

### Body Composition and Food Intake Measurement

The BM was measured every 4 weeks, between 8 am and 12 pm. Diet intake was calculated by the difference in weight between the amount of food offered subtracting the amount of food remaining. The energy intake per rat (kcal/week/rat) was calculated as: food consumption × Et (Et is the total energy of the diet which is 4.665 kcal/g in HFD and 3.854 kcal/g) [adapted from Gong et al. (2016)]. To assess body composition, the fed animals underwent anesthesia using an intra-peritoneal injection with ketamine (80 mg/kg) and xylazine (32 mg/kg), before euthanasia. Rats were later placed in prone position to be scanned using the DXA-Dual Range Emission Densitometry-between 8 am and 12 pm (Hologic Inc., Bedford, MA, United States). Thus, the body fat (BF), FM, fat-free mass (FFM) were obtained. Image analysis was performed using the QDR 4500 software (Hologic^®^).

### Experiments and Sample Collection

Visceral adipose tissue (EPI, RET, and MES) and gastrocnemius were dissected and weighed. The blood was obtained immediately after decapitation. Then, the head was removed and the neck was placed in a funnel attached to a collection tube without anticoagulant. The collection tube remained at RT for approximately 30 min until coagulation. After that, the samples were centrifuged at 1,500 × *g* for 10 min at 4°C to obtain the serum. The serum was then collected and placed in 500 μl aliquots in the Eppendorf and frozen at −80°C for further analysis.

### Glucose and Insulin Assessment

Blood glucose was measured using an Accu-Check glucometer (Roche Diagnostic, Indianapolis, IN, United States), after 12 h of fasting, immediately before euthanasia. A puncture was performed in the caudal vein of the animal to obtain blood. A drop of blood was placed on the edge of the test strip. After the capillary was filled, the device automatically showed the blood glucose value. The insulin was analyzed through an ELISA assay from the serum obtained after euthanasia. The insulin kit (ER1113) was purchased by Fine Biotech Co., Ltd. (Wuhan, China) and the test was performed according to the manufacturer’s instructions. The HOMA-IR index (model for assessing insulin resistance homeostasis) is a method that is based on plasma glucose and insulin and has been used to define insulin resistance (Matthews et al., 1985). To do this, the HOMA-IR index was calculated using the formula: fasting insulin (ng/ml) × fasting glycemia (mg/dl)/405 (Roza et al., 2016).

### Lipid Profile

Levels of high-density lipoproteins (HDL) (Ref. K015-Bioclin) and triglycerides (TG) (Ref. K117-Bioclin) were determined using the colorimetric enzymatic method (Bioclin, Belo Horizonte, Brazil), after 12 h of fasting. To do this, the serum was collected as previously described.

### Obesity-Related Inflammatory Markers

To assess the serum concentration of inflammatory and anti-inflammatory markers, an ELISA analysis was performed. The cytokines IL-1β (ab255730) and leptin (ab100773) were purchased by Abcam^®^ (Cambridge, United Kingdom and the cytokine IL-10 (no.555134) was purchased by BD Biosciences Pharmingen (San Diego, CA, United States) and, the myokine irisin (MET-5089) was purchased by Cell Biolabs (San Diego, CA, United States). The test was performed according to the manufacturer’s instructions.

### Protein Extraction and Western Blotting Analyses

Tissues were lysed with extraction protein buffer [SDS 0,1% (p/v); Triton 1% (v/v); Tris–HCl pH 7,8; 50 mM; NaCl 150 mM; EDTA 15 mM; EGTA 5 mM] as well as protease inhibitors (Complete-Mini Roche 1×). Equal amounts of each protein sample (60 μg) were separated by sodium dodecyl sulfate-polyacrylamide gel electrophoresis and transferred to polyvinylidene fluoride (PVDF) membranes (GE HealthCare, Marlborough, MA, United States). After blocking the membranes with 5% skim milk in TBST, the membranes were incubated with anti-FNDC5 antibody (1:1,000; ab174833, Abcam) and anti GAPDH (ab181602, Abcam/MAB5718, R&D System). Then, the membranes were incubated for 1h at room temperature with an appropriate secondary antibody: horseradish peroxidase (HRP)-conjugated goat anti-rabbit IgG (1:2,500, sc-2004, Santa Cruz); anti-rabbit (1:10,000, sc2357, Santa Cruz); anti-mouse (1:5,000, sc516102, Santa Cruz). Each protein band was visualized using an Amersham ECL Advance Western Blotting Detection Kit (GE Healthcare).

### Histopathological Analysis of Visceral Depots

For histopathological analysis, the EPI, RET and MES tissues were fixed in 10% formalin for 48 h. Dehydration was performed in increasing alcohol baths (90%, 100% I, 100% II, 100% III, and 100% IV) for periods of 60 min, diafinized in Xylol baths (50% Xylol I, 50% Xylol II, and 50% Xylol III) and finally embedded in paraffin (Merck Milipore). Tissues were cut into 5-μm sections and stained with HE. The images were digitized on a histological slide scanner (Panoramic Desk, 3DHISTECH Ltd., Hungary). To analyze the morphometry of the adipocyte images, five fields from each sample were collected with a 20× magnification (Panoramic Viewer) for quantification. The files were analyzed for the adipocyte area by the Adiposoft plugin (v. 1.15) from ImageJ Fiji (v 2.0.0). The Adiposoft plug-in was equalized with a diameter between 25 and 200 microns according to the program calibration. For each condition, a sample of *n* = 5 was selected and 100 fat cells per animal were evaluated (Ferland et al., 2020).

### Statistical Analysis

All statistical analyses were performed using Graph-Pad Prism Version 8.0 and R. To verify if the data followed a normal distribution, the Kolmogorov–Smirnov test was performed in each dataset. Data are presented as the mean ± SEM. At the first intervention, comparisons between groups were performed using a two-tailed Student’s *t*-test or Mann–Whitney *U* test depending on the normality of the data. The effect of diet and exercise and the interaction of training × diet, from the second intervention, was analyzed using two-way ANOVA. When ANOVA was not indicated, we used ANOVA-ART (aligned rank transform ANOVA) (Elkin et al., 2021). The Dunn or Tukey test (depending on the normality of the data) for *post hoc* analysis was performed to assess multiple comparisons. Spearman’s correlation coefficient was used to analyze the correlations between all study variables and the interpretation was performed according to Mukaka (2012). The criterion for statistical significance was *p* < 0.05 (two-tailed), using *p* values adjusted for multiple comparisons by false discovery rate (FDR). The data are presented as mean ± standard error (SEM).

## Results

### High-Fat Diet Promoted Obese Phenotype by Increasing Body Mass, Visceral Adiposity, and Serum Irisin Concentration

The body composition parameters at the end of the first intervention are described in Table 2. After 8 weeks of diet-induction obesity, a slight increase of ∼20% was observed in the BM of HFD animals. Additionally, all the visceral depots (EPI, RET, and MES) were significantly elevated in the HFD group compared to CD. As expected HFD had higher BF than CD animals. Regarding the biochemical parameters, irisin and glucose registered increased values in the HFD group. Irisin showed an almost threefold increase in the HFD group when compared to the CD group and on the other hand, FNDC5 was increased in the CD group compared to HFD. None of the cytokines, insulin and HOMA-IR differed significantly between the CD and the HFD group.

**TABLE 2:** Body composition of diet-induced obesity for 8 weeks.

Parameters	CD (*n* = 7)	HFD (*n* = 7)	*P*
BM (g)	509.1 ± 13.1 (6.8)	613.7 ± 13.7 (5.9)	<0.0001*
BF (%)	12.53 ± 0.71(15.1)	22.47 ± 1.33 (15.7)	<0.0001*
FFM (g)	445.0 ± 10.6 (6.3)	475.4 ± 8.0 (4.4)	0.0417*
FM (g)	64.0 ± 4.4 (18.2)	138.3 ± 10.5 (20.0)	<0.0001*
MES (g/100 g BM)	0.71 ± 0.06 (23.7)	1.73 ± 0.17 (26.0)	0.0001*
RET (g/100 g BM)	1.00 ± 0.11 (29.9)	2.13 ± 0.20 (25.2)	0.0004*
EPI (g/100 g BM)	1.14 ± 0.11 (26.9)	2.40 ± 0.24 (26.4)	0.0006*
Glucose (mg/dl)	100.9 ± 3.7 (9.8)	115.7 ± 3.2 (7.3)	0.0107*
Insulin (ng/ml)	0.28 ± 0.04 (37.2)	0.33 ± 0.04 (31.8)	0.2593
HOMA-IR	0.07 ± 0.01 (34.4)	0.10 ± 0.01 (31.1)	0.0855
TG	180.6 ± 24.2 (35.5)	236.2 ± 38.2 (42.8)	0.2423
HDL	41.8 ± 7.2 (45.5)	69.1 ± 8.7 (33.5)	0.0326*
Irisin (ng/ml)	3.02 ± 0.50 (43.5)	8.20 ± 2.3 (73.7)	0.0472*
IL-1β (pg/ml)	209.7 ± 19.0 (23.9)	201.6 ± 24.2 (31.8)	0.6200
IL-10 (pg/ml)	378.0 ± 23.9 (16.7)	351.6 ± 60.9 (45.9)	0.6940
Leptin (pg/ml)	145.8 ± 44.0 (79.8)	226.8 ± 74.5 (86.9)	0.3674
FNDC5 (relative density)	1.42 ± 0.01(18.2)	1.10 ± 0.06 (15.8)	0.0166*

CD: chow diet; HFD: high-fat diet BM: body mass; BF: body fat; FFM: fat-free mass; FM: fat mass; MES: mesenteric adipose tissue; RET: retroperitoneal adipose tissue; EPI: epididymal adipose tissue; HOMA-IR: Homeostatic model assessment; IL-1β: interleukin-1 beta; IL-10: interleukin-10. Results are means ± SEM (%CV). *p < 0.05 vs. control (*p*-values refer to the Mann–Whitney *U* test or Student *t*-test depending on the normality of the data).

### Epididymal Adipose Tissue Responds Later Than Mesenteric and Retroperitoneal Depots to Diet and Exercise Intervention

**FIGURE 2:**
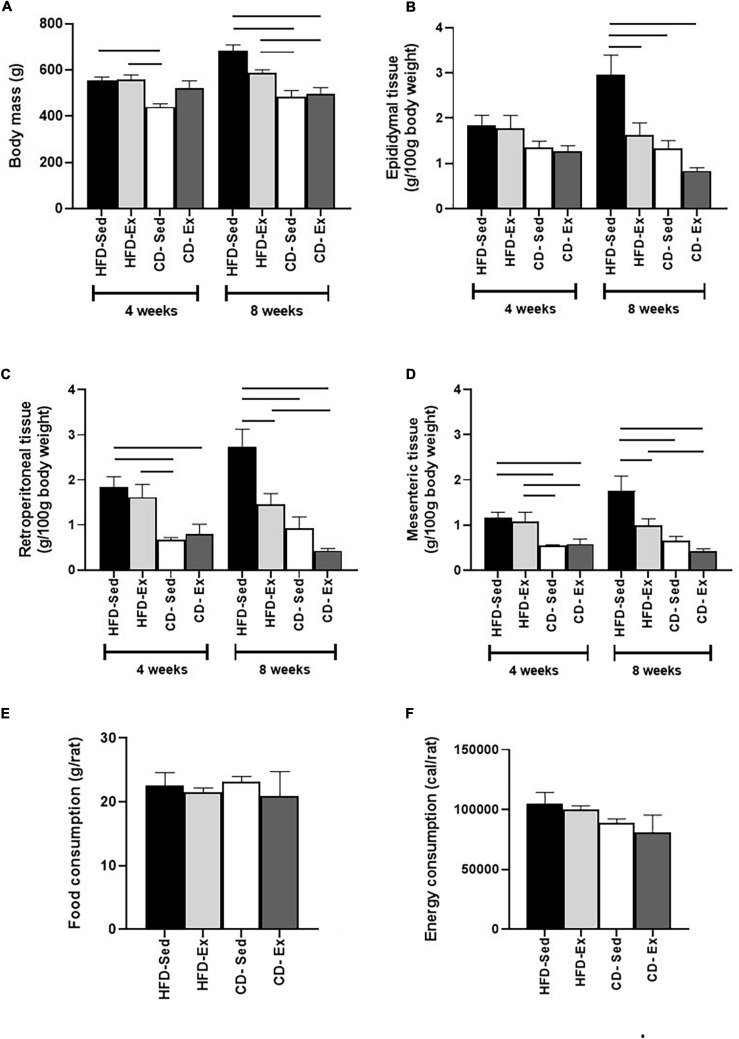
Effects of diet and exercise on body mass and visceral depots. **(A)** Body mass was assessed by DXA every 4 weeks. **(B)** Epididymal adipose tissue of male *Wistar* rats. **(C)** Retroperitoneal adipose tissue of male *Wistar* rats. **(D)** Mesenteric adipose tissue of male *Wistar* rats. **(E)** Food consumption of *Wistar* rats after 8 weeks of intervention. **(F)** Energy consumption of *Wistar* rats after 8 weeks of intervention. HFD-Ex: high-fat diet and exercise; HFD-Sed: sedentary high-fat diet; CD-Ex: chow diet and exercise; CD-Sed: sedentary chow diet. The bars represent the significant differences between the groups indicated. Results are means ± SEM (*p* < 0.05).

As shown in Figure 2A, at the beginning of the first intervention (4 weeks), the animals in the CD-Sed group (440.6 ± 13.1 g, *p* < 0.001) showed reduced BM when compared to the HFD groups. However, after 8 weeks, a significant difference was observed in the two CD groups when compared to the two HFD groups.

Visceral fat depots were also assessed during the experimental protocol, as represented in Figures 2B–D. We observed a similar response from RET and MES depots to diet and training interventions. In the fourth week, the CD groups had a reduction in RET and MES compared to the HFD groups. At the end of the 8th week, CD-Ex, CD-Sed and HFD-Ex showed a reduction in RET and MES compared to the HFD-Sed group. As shown in Figure 2B, no differences in EPI tissue mass were found after 4 weeks of intervention. However, after 8 weeks, the HFD-Ex group and the two CD groups showed a reduction in relation to HFD-Sed (2.95 ± 0.54 g/100 g BM).

Food and energy consumption was also assessed throughout the period and are shown in Figures 2E,F. There was no difference in food and energy consumption.

**FIGURE 3:**
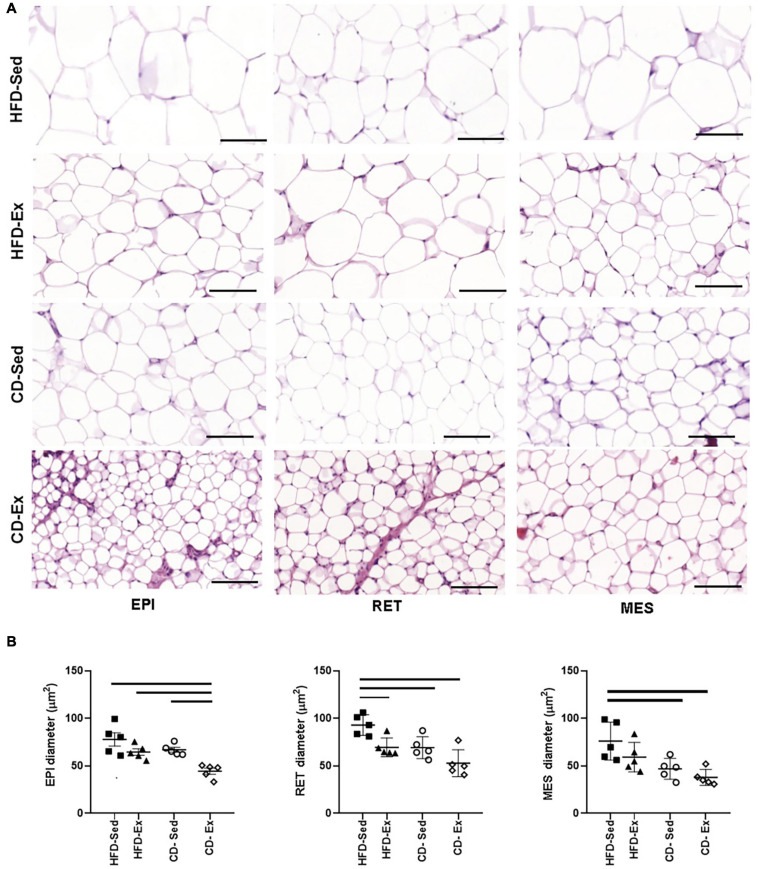
Photomicrograph of adipose tissue after 8 weeks of dietary intervention and training. **(A)** Representative images of adipose tissue showing hematoxylin and eosin staining using 20× (100 μm) objective, light-field microscopy. **(B)** Diameter of EPI, RET and MES adipocytes; RET: retroperitoneal adipose tissue; MES: mesenteric adipose tissue. HFD-Ex: trained high-fat diet group; HFD-Ex: high-fat diet and exercise; HFD-Sed: sedentary high-fat diet; CD-Ex: chow diet and exercise; CD-Sed: sedentary chow diet. The bars represent the significant differences between the indicated groups. The results are presented as means ± SEM (*p* < 0.05).

Regarding the diameter of the adipocytes (Figure 3), a similar behavior was observed in the RET and MES depots. The lowest values of the EPI diameter were observed in the CD-Ex animals (44.49 ± 3.19 μm) when compared to all other groups. In RET, the highest values of diameter were observed in the animals HFD-Sed (92.95 ± 4.84 μm) in relation to all groups. In the MES tissue, the largest area record was observed in the animals in the HFD-Sed groups (75.81 ± 8.83 μm) in relation to the groups fed the standard diet.

Following the characterization of Verboven et al. (2018) adipocytes were classified as small (< 50 μm), medium (50–69 μm), large (70–89 μm), and very large (>90 μm) (Verboven et al., 2018). The HFD-Sed animals had in the three visceral deposits large adipocytes (>75 μm), however in the RET tissue, the adipocytes were classified as very large. The animals in the HFD-Ex group had mean adipocytes in the three compartments, similar to the diameter of the CD-Sed animals. CD-Sed animals also showed small adipocytes in the MES tissue. The CD-Ex group, on the other hand, showed a reduction in all adipocytes and only adipocytes classified as small and medium were observed.

### Short-Term Diet Intervention Reduces Visceral Adiposity and Fat-Free Mass, While Short-Term Exercise Only Increases Fat-Free Mass in Animals With Improved Metabolic Profile

**FIGURE 4:**
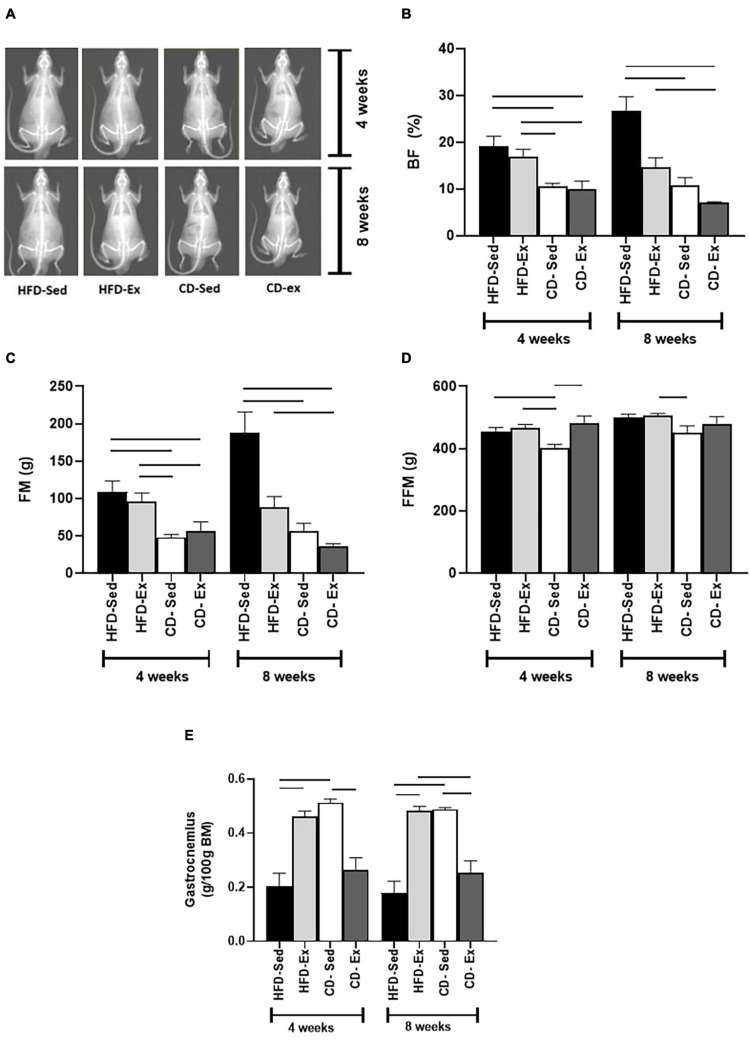
Effects of diet and exercise on body composition. **(A)** DXA images of high-fat diet (HFD) and chow diet (CD) groups. **(B)** Body fat (%) was assessed after 4 and 8 weeks. **(C)** Fat mass was determined at weeks 4 and 8. **(D)** Fat-free mass was registered every 4 weeks. **(E)** Gastrocnemius mass was assessed at 4 and 8 weeks. The bars represent the significant differences between the groups indicated. HFD-Ex: high-fat diet and exercise; HFD-Sed: sedentary high-fat diet; CD-Ex: chow diet and exercise; CD-Sed: sedentary chow diet. The bars represent the significant differences between the indicated groups. Results are means ± SEM (*p* < 0.05).

To determine the effects of diet and exercise on adiposity, BF and FM were assessed by DXA (Figure 4). FM and BF had a similar response. After 4 weeks, CD-Sed and CD-Ex had lower BF and FM than HFD-Ex and HFD-Sed. At the end of the experiment (8 weeks), the CD-Ex and CD-Sed remained with reduced values compared to the HFD-Sed group (BF: 26.73 ± 3.00%; FM: 187.6 ± 28.16 g, *p* < 0.05). The short-term effects of interventions in FFM (4 weeks) (Figure 4D) showed that the lowest values were observed in the CD-Sed group (403.30 ± 10.81 g) compared to the HFD-Ex, HFD-Sed and CD-Ex groups. At the end of the experiment, the only difference was observed in the CD-Sed group compared to the CD-Ex group.

After 4 weeks, the gastrocnemius muscle (Figure 4E) was elevated in the HFD-Ex and CD-Sed groups compared to the HFD-Sed and CD-Ex/HFD-Sed groups, respectively. After 8 weeks, the same results were repeated and the HFD-Sed group had a lower gastrocnemius mass compared to the HFD-Ex and CD-Sed group, and the CD-Ex group had a reduced gastrocnemius mass compared to the CD-Sed and HFD-Ex.

### The Exclusive Diet Was the Most Important Factor to Mitigate the Biochemical and Inflammatory Markers Related to Obesity

**FIGURE 5:**
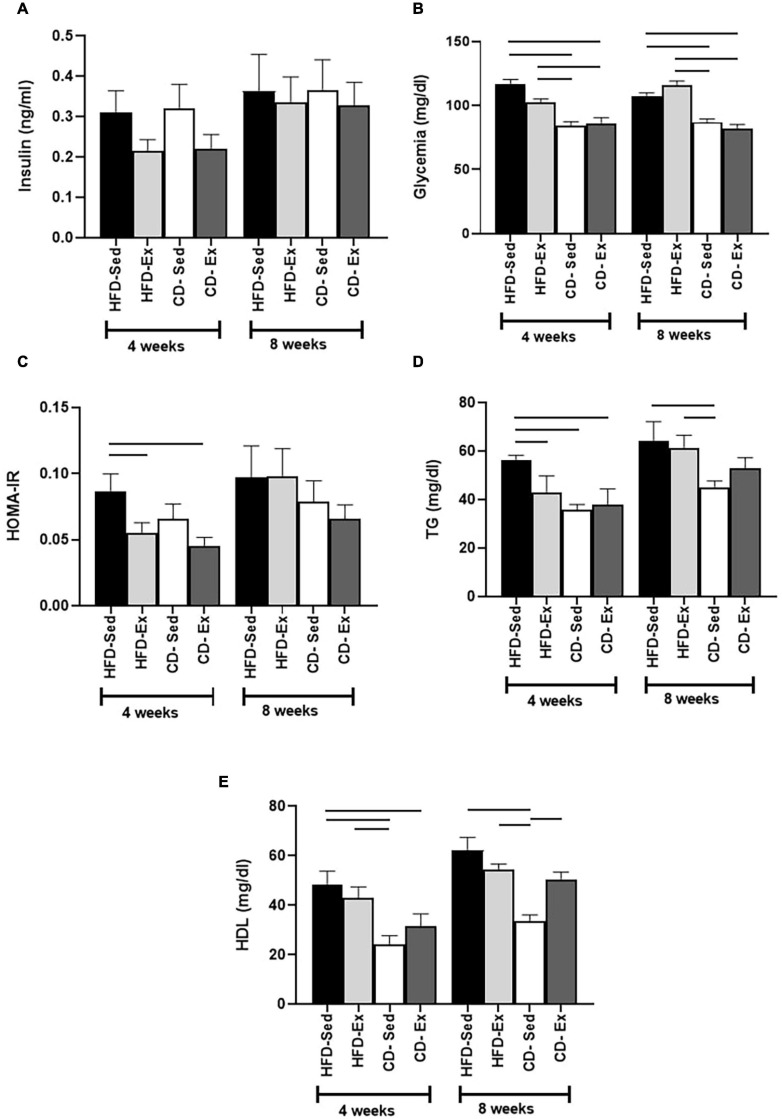
Effects of diet and exercise on body composition on biochemical parameters. **(A)** Serum insulin concentration after 4 and 8 weeks of dietary and training interventions. **(B)** Blood glucose after 4 and 8 weeks of dietary and training interventions. **(C)** HOMA-IR index after 4 and 8 weeks of dietary and training interventions. **(D)** Serum triglycerides levels after 4 and 8 weeks of dietary and training interventions. **(E)** High-density lipoproteins (HDL) levels after 4 and 8 weeks of dietary and training interventions. HFD-Sed: sedentary high-fat diet; CD-Ex: chow diet and exercise; CD-Sed: sedentary chow diet. The bars represent the significant differences between the groups indicated. Results are means ± SEM (*p* < 0.05).

The glycemic and lipid profile was also assessed in this study (Figure 5). There were no significant differences in serum insulin levels between the groups after 4 and 8 weeks (Figure 5A). Both control diet groups showed a significant reduction in glycemia compared to the HFD groups after 4 and 8 weeks of intervention (Figure 5B). We also evaluated the HOMA-IR (Figure 5C) index between the groups. We observed that significant differences appeared only after 4 weeks of intervention. The HFD-Sed group (0.09 ± 0.01) showed higher values compared to the CD-Ex and HFD-Ex groups.

Regarding lipid fractions, TG levels (Figure 5D) increased in the HFD groups after 4 and 8 weeks of intervention. The HFD-Sed group (56.11 ± 2.22 mg/dl) presented the highest values in the fourth week, compared to the HFD-Ex, CD-Sed and CD-Ex groups. In the eighth week, the CD-Sed group (45.14 ± 2.55 mg/dl) had the lowest values, which were significantly different from the HFD-Sed and HFD-Ex groups.

Regarding the HDL fraction (Figure 5E), in the fourth week, it was observed that the CD-Sed animals (24.31 ± 3.38 mg/dl) registered the lowest values and were significantly different from HFD-Ex and HFD-Sed. The CD-Ex group (31.71 ± 4.87 mg/dl) also showed a reduction in values compared to the HFD-Sed group but did not differ from the HFD-Ex group. After 8 weeks, the response of HDL was similar to the response of the fourth week, the CD-Sed group (33.42 ± 2.70 mg/dl) showed the lowest values in relation to the animals of the groups HFD-Sed, HFD-Ex, and CD-Ex.

### Diet and Exercise Exclusively Were the Most Important Factors in the Modulation of Irisin/FNDC5

**FIGURE 6:**
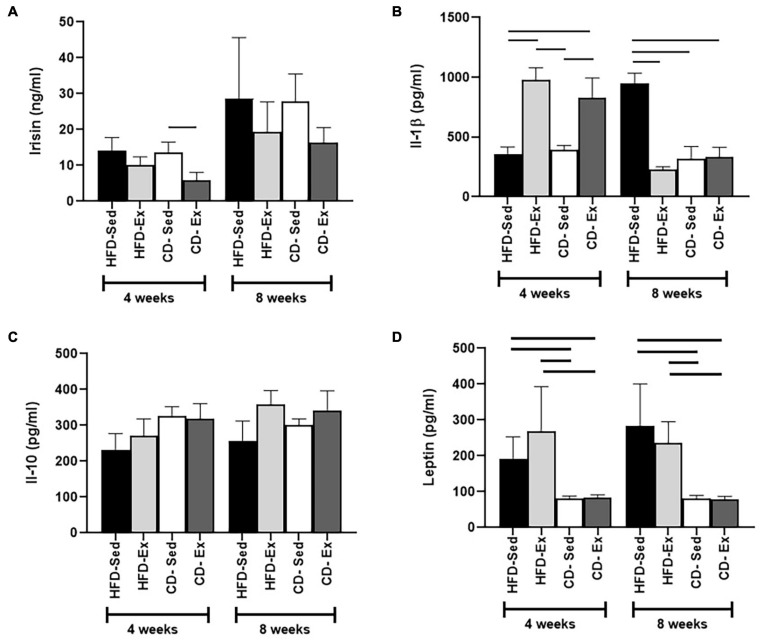
Determination of serum levels of adipocytokines during the experiment. **(A)** Serum irisin concentration after 4 and 8 weeks of dietary and training interventions. **(B)** Interleukin 1-beta (IL-1β) was evaluated every 4 weeks. **(C)** Levels of interleukin 10 (IL-10) were determined after 4 and 8 weeks. **(D)** Serum leptin levels after 4 and 8 weeks.

Changes in irisin were observed only after 4 weeks of intervention, but only in the CD groups (Figure 6A). The CD-Sed group (13.57 ± 3.00 ng/ml) registered higher values than the CD-Ex group (5.81 ± 2.30 ng/ml), which registered the lowest serum concentrations. Interestingly, after 8 weeks there were no significant differences between groups.

Serum IL-1β values were determined by ELISA (Figure 6B). Interestingly, 4 weeks after the intervention, reduced cytokine values were observed in the HFD-Sed group (359.3 ± 58.5 pg/ml) compared to the HFD-Ex and CD-Ex groups. In addition, the CD-Sed group (391.0 ± 39.6 pg/ml) also showed a reduction in IL-1β compared to the HFD-Ex and CD-Ex groups (Figure 6B). However, at week 8, the animals in the HFD-Sed group (948.2 ± 85.6 pg/ml) registered the highest values compared to the other groups. The IL-10 values (Figure 6C) showed that there was no significant difference between the groups after the 4 and 8 weeks of intervention. In the initial weeks (4 weeks) and after 8 weeks of intervention, the leptin values showed the same behavior. Both control diet groups showed a significant reduction in serum leptin in relation to the HFD groups after 4 and 8 weeks of intervention (Figure 6D).

**FIGURE 7:**
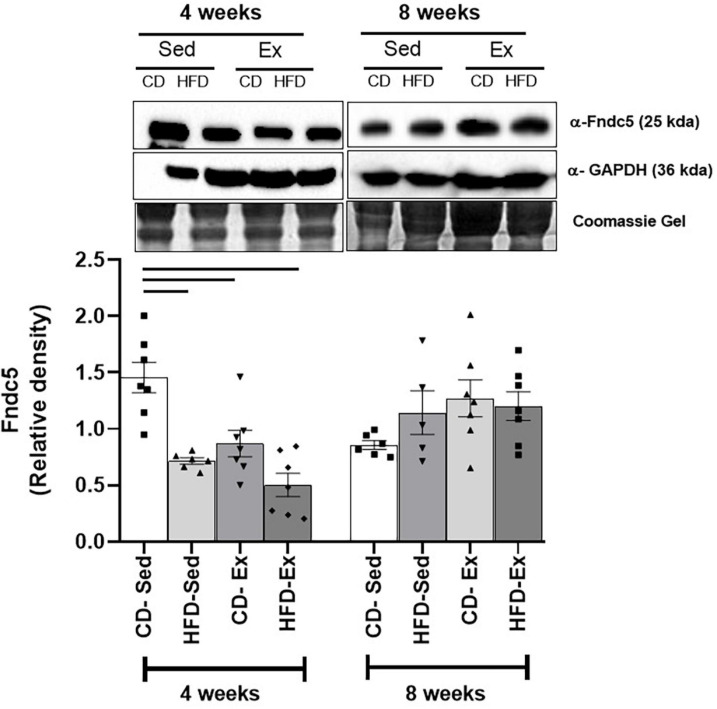
Protein expression of FNDC5. The presence of a 25 kDa band was detected in the gastrocnemius with anti-FNDC5 antibodies after 4 and 8 weeks of dietary intervention and training; GAPDH was used as a loading control. HFD-Ex: high-fat diet and exercise; HFD-Sed: sedentary high-fat diet; CD-Ex: chow diet and exercise; CD-Sed: sedentary chow diet. The bars represent the significant differences between the indicated groups. The results are presented as means ± SEM (*p* < 0.05).

Protein expression was assessed in the gastrocnemius muscle after 4 and 8 weeks of dietary intervention and training (Figure 7). After 4 weeks, higher values were observed in the CD-Sed group (1.45 ± 0.14) compared to all other groups HFD-Sed, HFD-Ex, and CD-Ex. No significant differences were observed after 8 weeks of intervention.

**FIGURE 8:**
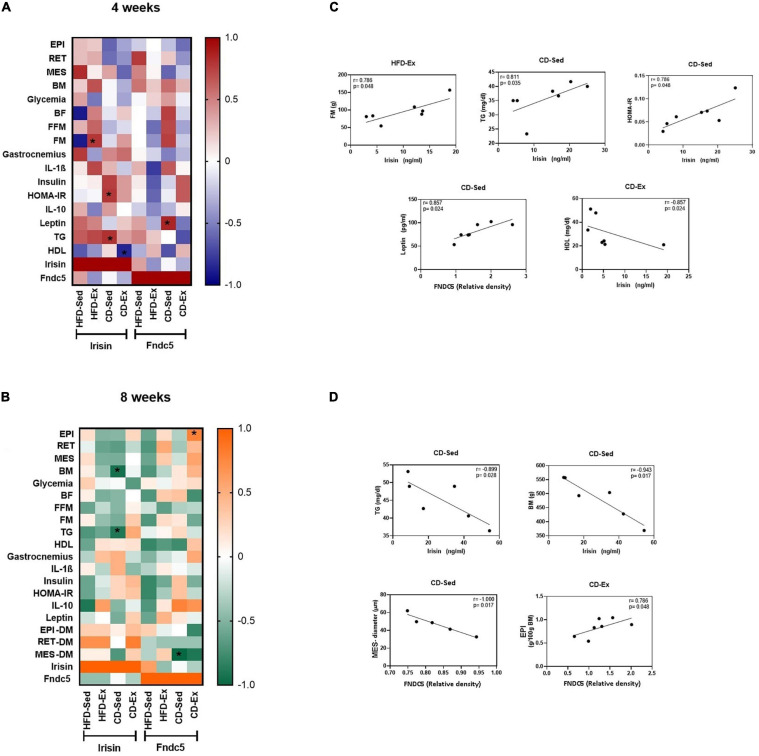
Spearman’s correlation coefficient between irisin/FNDC5, body composition and biochemical parameters after 4 and 8 weeks of intervention. **(A)** Heatmap of Spearman’s correlation coefficient between irisin/FNDC5, body composition and biochemical parameters after 4 weeks of intervention. **(B)** Heatmap of Spearman’s correlation coefficient between irisin/FNDC5, body composition and biochemical parameters after 8 weeks of intervention. **(C)** Spearman’s correlations that were significant between irisin/FNDC5 and the study variables after 4 weeks of intervention. **(D)** Spearman’s correlations that were significant between irisin/FNDC5 and the study variables after 8 weeks of intervention. EPI: epididymal adipose tissue; RET: retroperitoneal adipose tissue; MES: mesenteric adipose tissue; BM: body mass; BF: body fat; FFM: fat-free mass; FM: fat mass; HOMA-IR: Homeostatic model assessment; TG: triglycerides; HDL: high density lipoprotein; IL-1β: interleukin 1-beta; IL-10: interleukin 10; AR: area; DM: diameter. HFD-Ex: high-fat diet and exercise; HFD-Sed: sedentary high-fat diet; CD-Ex: chow diet and exercise; CD-Sed: sedentary chow diet. Individual values were plotted on the graphs. Statistical significance: *p* < 0.05.

To verify whether there was a correlation among the metabolic parameters, the body composition and irisin/FNDC5, a heatmap was designed and Spearman’s correlation was performed and analyzed (Figure 8). After the 4 weeks of intervention, there was a positive correlation in the animal HFD-Ex between irisin and FM (*r* = 0.786/*p* = 0.048); in CD-Sed animals between irisin and TG (*r* = 0.811/*p* = 0.035); between irisin and HOMA-IR (*r* = 0.786/*p* = 0.048) and between FNDC5 and leptin (*r* = 0.857/*p* = 0.024). In CD-Ex animals, a negative correlation was observed between irisin and HDL (*r* = −0.857/*p* = 0.024). After 8 weeks, significant correlations were found in the CD-Sed group, a positive and strong correlation between irisin and BM (*r* = −0.943/*p* = 0.017) and a strong and negative correlation between irisin and TG (*r* = −0.899/*p* = 0.028); and between the FNDC5 and MES diameter (*r* = −1,000/*p* = 0.017). A strong positive correlation was observed between FNDC5 and EPI (*r* = 0.786/*p* = 0.048).

## Discussion

Diet and physical inactivity play a major role in the genesis of obesity and irisin/FNDC5 modulation. Here we report that the diet was effective in promoting an obese phenotype after 8 weeks, including augmentation in body adiposity, BM, and visceral depots. These findings support previous studies that used a similar composition to induce obesity after six (Oishi et al., 2018) and eight weeks (Estadella et al., 2004). In this study, 8 weeks after the introduction of HFD an increase in irisin secretion was noted. This observation supports the theory that irisin plays a compensatory role during metabolic disorders, such as obesity, impaired glucose homeostasis and insulin resistance (Guilford et al., 2017). Interestingly, contrary to the values of irisin, the expression of FNDC5 in the gastrocnemius muscle was shown to be significantly elevated in the diet intervention group. Although the findings show that the highest expression of FNDC5 occurs through muscle (in physiological or pathological conditions), it has been reported that irisin is partly secreted by VAT in Frühbeck et al. (2020), Kirat et al. (2021). This suggests that the origin of FNDC5, which increased the circulating irisin values in HFD animals, may be of adipose origin.

Long-term exposure to HFD resulted in the obesity phenotype, with an increase in all visceral depots, BF and FM. The dietary intervention was responsible for affecting most of the parameters evaluated. The exercise had an influence on FFM on short-term and, visceral adiposity in long-term intervention. In contrast, irisin/FNDC5 was affected by diet and exercise exclusively, but not by their combination. Diet and training were proposed as interventions in obese rats in this study to observe the modulation of irisin/FNDC5. We demonstrated that after 4 weeks, exercise was the most important factor in reducing irisin secretion. Moreover, high-fat diet and exercise were the most important factors in reducing FNDC5 secretion. The association between chronic consumption of HFD has been explored in the literature and there is no consensus on the findings. In humans, irisin was highly and positively related to BMI, BM and BF (Park et al., 2013; Crujeiras et al., 2014; Pardo et al., 2014; Fagundo et al., 2016; Sahin-Efe et al., 2018). However, in rodents, several effects have been reported. Quiñones et al. (2015) showed that Sprague-Dawley mice that received HFD for 10 weeks did not detect any significant difference in irisin levels compared to control (Quiñones et al., 2015). Kang et al. (2019) showed that Sprague-Dawley mice that received HFD for 16 weeks presented a reduction in irisin levels at the end of the experiment (Kang et al., 2019). The same findings were reported by Lu et al. (2016) after 24 weeks of HFD and Yang et al. (2015) in C57BL/6 mice after 12 weeks of HFD (Yang et al., 2015; Lu et al., 2016). Subsequently, data showed that this decline did not influence the BM and glucose that were increased during the experiment. In these animals, the reduction was probably due to adipose FNDC5 since the concentrations of irisin in the skeletal muscle remained unchanged. Positive associations between irisin and obesity were demonstrated by Guilford et al. (2017), who reported that mRNA *Fndc5* in adipose tissue was significantly higher in HFD compared to counterparts (Guilford et al., 2017). Moreover, they were reinforced by Kazeminasab et al. (2018) who observed the highest irisin secretion in obese mice, compared to non-obese mice (Liu et al., 2017). In line with the earlier findings in rodents, we did not observe an increase in the expression of irisin/FNDC5 in animals fed a HFD, but only in CD-Sed animals. In addition, a significant improvement in glycemic and lipid profile, besides a reduction in visceral adiposity was observed. Thus, we can attribute the highest secretions of irisin to a more favorable glycemic/adipose profile. This observation does not support the theory that irisin plays a compensatory role during metabolic disturbances such as obesity, impairments in glucose homeostasis, and insulin resistance (Roca-Rivada et al., 2013; Guilford et al., 2017).

Thus, blood glucose was assessed in this study. Our data showed evidence of a significant increase in blood glucose in both the HFD groups after 4 and 8 weeks when compared to CD animals. However, these changes are not due to exercise-training exclusively. Irisin appears to play an active role in improving glucose homeostasis and higher levels suggest a therapeutic potential in the control of comorbidities associated with insulin resistance (Moreno-Navarrete et al., 2013). *In vitro*, irisin increases glucose uptake by muscle cells by p38 AMPK pathway promoting the proliferation of β cells (Boström et al., 2012; Lee et al., 2015; Yang et al., 2015). Moreover, irisin prevents apoptosis of pancreatic β cells resulting from persistent hyperglycemia, through negative regulation of pro-apoptotic proteins (Liu et al., 2017). Data *in vivo* confirmed that irisin increased glucose uptake by stimulating GLUT4 translocation in skeletal muscle cells of HFD-mice treated with exogenous irisin (Huh et al., 2014; Lee et al., 2015). Obese and diabetic/non-diabetic individuals that had increased secretion of irisin may be due to an attempt to improve glucose uptake and to prevent hyperglycemia (Perakakis et al., 2017). The response of reduced levels of glycemia in the diet intervention group that showed improvements in serum blood glucose levels, reinforcing the associations of glycemic profile and the action of irisin/FNDC5. However, this statement is only relevant in the short term and is not supported in the long-term interventions, therefore it may be a transitory mechanism and needs further investigations.

Maintenance of BM through diet and exercise training is beneficial in the prevention of metabolic disturbances associated with obesity (Fagundo et al., 2016; Verheggen et al., 2016). Initially, irisin was reported as an exercise-inducible myokine capable of mediating the beneficial effects of exercise and increasing thermogenesis, which contributes to maintaining energetic homeostasis (Boström et al., 2012). Since then, diverse endurance exercises have evaluated irisin circulation in humans and animals. Circulating levels of irisin are increased in individuals involved in exercise-induced activities and progressively reduced in those less active and sedentary (Arhire et al., 2019). Several studies show that short-term aerobic exercises (Anastasilakis et al., 2014; Aydin et al., 2014) and long-term exercises (Boström et al., 2012; Kim et al., 2016) upregulate FNDC5 and irisin levels in humans and animals promoting pleiotropic effects. Contradictory to those findings, some human studies failed to confirm the response of *Fndc5* mRNA and irisin by exercise (Hecksteden et al., 2013; Norheim et al., 2014; Hew-Butler et al., 2015). These discrepancies can be explained by the variability between species, exercise intensity, frequency, session duration, nutritional status, and training protocol (Fatouros, 2018). Recent data showed that in humans, training is often carried out that includes moderate to high intensity running (60–90%) with durations between 3 and 21 weeks of 2–3 days a week and showed controversial results (Fatouros, 2018). Our findings did not support the initial hypothesis that irisin is upregulated during exercise. Our data corroborate with human studies and here we show that irisin and FNDC5 were reduced in exercised animals compared to sedentary animals after 4 weeks of intervention, only in the diet intervention group. Part of our findings can be explained because the gastrocnemius muscle of the exercised control animals had a significantly reduced mass in relation to their non-exercised counterparts, however there was no significant correlation between these two variables. On the other hand, these discrepancies can be explained by the variability between species. In humans, frequently the protocols used to evaluate irisin and which failed to show an increase in circulating irisin were performed 2–3 times a week, while most animal studies used it 5 times a week (Fatouros, 2018). Therefore, our findings may reflect the chosen protocol that mimicked human training conditions. Our studies are corroborated by previous findings that show that in treadmill protocols, the results were not so unanimous. Continuous exercise and HIIT protocols increased serum irisin values compared to sedentary controls (Khalafi et al., 2020); however, some data showed that irisin was not changed after 8 weeks of training (Kazeminasab et al., 2018).

Diet plays an essential role in the genesis of obesity and metabolic syndrome, but the composition of the diet does not seem to directly interfere in the secretion of irisin. Anastasilakis et al. (2014) showed that the total caloric or macronutrient intake: carbohydrates, proteins, fats and fibers are not related to irisin. Nonetheless, De Macedo et al. (2017) demonstrated that mice fed high-fat (20% fat), high-carbohydrate (80% carbohydrate) diets for 60 days, had less expression of FNDC5 and irisin in the soleus muscle when compared to standard diet and high-protein diet (31% protein), respectively. In our findings, the diet exclusively seems to determine the serum concentrations of FNDC5 and, high levels were observed in CD-Sed animals compared to the groups fed a HFD. Our data are not supported by previous studies. In both human and mice showed that irisin/FNDC5 is decreased in response to a hypocaloric diet and caloric restriction (Crujeiras et al., 2014; Varela-Rodríguez et al., 2016). It is important to highlight that although we did not perform caloric restriction, a common feature of these interventions is the reduction of visceral adiposity, which was demonstrated here in this study, and which may have contributed to the increase in the secretion of irisin. The hypocaloric diet when not combined with exercise effectively induces weight loss but also reduces FFM (Willoughby et al., 2018). Since irisin is mostly released by muscle, a reduction in FFM may impair irisin secretion (Arhire et al., 2019). Supporting this hypothesis, a positive correlation between irisin and FFM has been reported (Stengel et al., 2013; Pardo et al., 2014). Our data partially corroborate the above, because CD-Sed animals showed a FFM reduction, however we can associate the high FNDC5/irisin values of CD-Sed animals with an elevation in gastrocnemius mass, as mentioned. Thus, the low levels of FNDC5 observed in HFD-Sed animals, can be attributed to a lower gastrocnemius mass. Low levels of circulating irisin have been reported in individuals with loss of muscle strength and atrophy (Chang et al., 2017). It is important to highlight that recent findings have shown that there is a significant interaction between the FNDC5 genotype and the state of sarcopenia in patients with non-alcoholic liver disease (Gao et al., 2020). Thus, irisin is a potential biomarker for muscle dysfunction and can help in the early diagnosis of sarcopenia and muscle changes associated with age (Chang et al., 2017).

Skeletal muscle represents the main source of secretion, with an expression of ∼72% of the total circulating levels of the protein (Boström et al., 2012). Additionally, FNDC5/irisin is secreted by adipocytes and is modulated in a manner dependent on the location of the fat depots (Roca-Rivada et al., 2013). Visceral depots appear to be greater contributors to circulating levels of irisin in rodents than subcutaneous adipocytes. VAT is a heterogeneous tissue with significant differences between regional depots. EPI adipocytes have more mitochondria, higher cytochrome oxidase, citric synthase activities and higher respiration rate than inguinal depots. RET adipose tissue had higher mRNA levels of lipolysis and lipogenesis-related genes than inguinal and mesenteric adipose tissue (Deveaud et al., 2004; Wronska and Kmiec, 2012; Chusyd et al., 2016; Schoettl et al., 2018). Most studies address the secretion of irisin against subcutaneous and visceral depots. This research investigated the relationship between irisin and different visceral compartments. Our findings indicate that after 4 weeks, circulating irisin was increased in CD-Sed animals and occurred synergistically to the reduction of visceral depots. However, we did not observe a significant correlation between irisin and BF, EPI, RET and MES after 4 weeks. Furthermore, the morphometry of visceral depots did not show a correlation with irisin/FNDC5 after 4 weeks. This may suggest that protein is mostly secreted by muscle tissue instead of adipose tissue even in obese rats.

We observed in the CD-Sed group, an increase in FNDC5 that was positively correlated with leptin. Our findings corroborate with previous studies that showed that leptin positively regulated FNDC5 expression in murine C2C12 myocytes and stimulated baseline myogenesis and lower mRNA expression of factors related to muscle atrophy (Rodríguez et al., 2015). In addition, in the CD-Sed group, an increase in serum irisin was observed, which was positively correlated with the HOMA-IR index. These results have been observed previously in obese men and women (Fukushima et al., 2016), in men and women independent of nutritional status (Park et al., 2013) and in patients with kidney diseases (Ebert et al., 2014). In contrast, in female children irisin correlated negatively with the HOMA-IR index (Al-Daghri et al., 2016), as well as in obese men (Moreno-Navarrete et al., 2013). Park et al. (2013) also showed that metabolic syndrome indicators were positively correlated with irisin, even when adjusted for BMI and BF. Our data corroborate the findings and there was a positive correlation between irisin and TG in the CD-Sed animals. This same correlation has already been observed in overweight non-diabetic individuals, however the correlation was weak and the other anthropometric markers related to the risk of cardiovascular events were negatively related to irisin (Tang et al., 2019). Moreover, in the CD-Ex animals of this study, a negative correlation between irisin and HDL was observed, previously reported by Huh et al. (2012) in obese and non-obese individuals (Huh et al., 2012). Although HDL values are not significantly elevated in CD-Ex animals compared to CD-Sed, there is a tendency for this increase, which may have contributed to the reduction of circulating irisin in these animals. Together these data reinforce that the values of irisin/FNDC5 are regulated differently both in the presence of exercise and nutritional status. We showed that the positive relationship with the inflammatory marker and TG and negative with HDL is only significant while the nutritional status of the animal is with reduced adiposity, since the same findings were not confirmed in animals fed a sedentary HFD.

International guidelines often recommend combining exercise and low-calorie diets to treat obesity (Verheggen et al., 2016; Kahn et al., 2019). Thus, it is relevant to understand the combined and isolated effects of each intervention, on visceral adiposity, and on irisin. The results of this investigation showed that diet exclusively induced beneficial changes most of the parameters related to visceral adiposity both short and long term, and had no effect on the serum concentrations of irisin, but downregulated FNDC5 expression. In 4 weeks, the diet reduced most of the parameters related to body adiposity, and this effect was enhanced in 8 weeks. In addition, the diet reduced the mass of all visceral depots after 4 and 8 weeks of intervention. On the other hand, the training independently reduced visceral depots only after 8 weeks of intervention. Regarding the body composition parameters evaluated, training did not have an effect on any of those. Contrary to what was hypothesized, although the combination of exercise and diet reduced the adiposity and inflammatory parameters of obesity, these effects were not enhanced by this combination but there is a tendency for that. Therefore, we reinforce the importance of combining the diet associated with exercise in the control of obesity, especially in the preservation of muscle mass. It is important to emphasize that the combination promoted effects superior to exercise in body composition, but did not overcome the effects of diet.

There are some limitations that should be noted when interpreting these results. First, our study sample size was relatively small and some data showed very heterogeneous values, limiting our power to detect differences between groups. Secondly, we were unable to identify the detailed source of irisin secretion into the visceral depots due to the absence of expression protein in the tissue sample. Lastly, though the scope of this current study was the interactions between irisin and visceral depots, it is important to highlight that muscle and adipose tissue are the major but not the only source of irisin in the body. We did not study SAT or heart–two other significant sources of irisin. These tissues have an important influence on the metabolism of adipocytes and may be responsible for part of the effects presented in that study.

In conclusion, considering the metabolic repercussions of obesity, VAT has been the focus of several studies that aim to attenuate the metabolic repercussions of obesity. Thus, new myokines, such as irisin, which actively participate in thermogenic regulation have been investigated. In this study, the diet was the most important factor in reducing visceral adiposity in the short and long term, followed by the combination of exercise and diet. Exercise was also important, as it preserves lean muscle mass and reduces visceral depots, after the diet. And diet and exercise exclusively were the factors capable of increasing the values of irisin, however, it did not bring cumulative effects of both interventions. Prescriptions to enhance the obesity treatments should involve reducing visceral adiposity as the focus of planning. To do this, reducing the fat content in the diet and aerobic exercise should be included as an initial treatment strategy. Furthermore, in addition to monitoring the classic biomarkers associated with obesity, such as blood glucose and HDL, irisin should also be evaluated as an early metabolic marker of obesity and FNDC5 as a marker of sarcopenia.

## Data Availability Statement

The original contributions presented in the study are included in the article/supplementary material, further inquiries can be directed to the corresponding author.

## Ethics Statement

Experimental protocols were approved by the Ethics Committee on the Use of Animals (no. 7631210617) at the Federal University of São Carlos (UFSCar).

## Author Contributions

VF, DM, AD, MS-F, JA, and CC helped to conceive the design, analyzed the data, and wrote the first draft of the manuscript. VF, DM, AD, JA, CA-L, SM, MS-F, RB, CC, CR, IM, and MR performed the other data analysis and helped to draft the manuscript. VF, DM, JA, MS-F, AD, and CR helped to conceive the design, and supervised the experimental trials and training sessions. VF, CC, AD, MS-F, CR, DM, JA, RB, IM, and MR interpreted the study results and edited the manuscript. VF, CC, AD, CA-L, SM, MS-F, CR, DM, JA, RB, IM, and MR helped to conceive the design, assisted with data analyses, provided funding for the study, and helped to draft the manuscript. All authors contributed to the article and approved the submitted version.

## Conflict of Interest

The authors declare that the research was conducted in the absence of any commercial or financial relationships that could be construed as a potential conflict of interest.

## Publisher’s Note

All claims expressed in this article are solely those of the authors and do not necessarily represent those of their affiliated organizations, or those of the publisher, the editors and the reviewers. Any product that may be evaluated in this article, or claim that may be made by its manufacturer, is not guaranteed or endorsed by the publisher.

## References

[B1] Al-Daghri N. M. Mohammed A. K. Al-Attas O. S. Amer O. E. Clerici M. Alenad A. (2016). SNPs in FNDC5 (Irisin) are associated with obesity and modulation of glucose and lipid metabolism in Saudi subjects. *Lipids Health Dis.* 15:54. 10.1186/s12944-016-0224-5 PMC4788945 26968837

[B2] Anastasilakis A. D. Polyzos S. A. Saridakis Z. G. Kynigopoulos G. Skouvaklidou E. C. Molyvas D. (2014). Circulating irisin in healthy, young individuals: day-night rhythm, effects of food intake and exercise, and associations with gender, physical activity, diet, and body composition. *J. Clin. Endocrinol. Metab.* 99 3247–3255. 10.1210/jc.2014-1367 24915120

[B3] Arhire L. I. Mihalache L. Covasa M. (2019). Irisin: a hope in understanding and managing obesity and metabolic syndrome. *Front. Endocrinol. (Lausanne)* 10:524. 10.3389/fendo.2019.00524 31428053 PMC6687775

[B4] Aydin S. Kuloglu T. Aydin S. Eren M. N. Celik A. Yilmaz M. (2014). Cardiac, skeletal muscle and serum irisin responses to with or without water exercise in young and old male rats: cardiac muscle produces more irisin than skeletal muscle. *Peptides* 52 68–73. 10.1016/j.peptides.2013.11.024 24345335

[B5] Bonfante I. L. P. Chacon-Mikahil M. P. T. Brunelli D. T. Gáspari A. F. Duft R. G. Oliveira A. G. (2017). Obese with higher FNDC5/irisin levels have a better metabolic profile, lower lipopolysaccharide levels and type 2 diabetes risk. *Arch. Endocrinol. Metab.* 61 524–533. 10.1590/2359-3997000000305 29412381 PMC10522056

[B6] Boström P. Wu J. Jedrychowski M. P. Korde A. Ye L. Lo J. C. (2012). A PGC1a dependent myokine that derives browning of white fat and thermogenesis. *Nature* 481 463–468. 22237023 10.1038/nature10777 PMC3522098

[B7] Bray G. A. Kim K. K. Wilding J. P. H. (2017). Obesity: a chronic relapsing progressive disease process. A position statement of the World Obesity Federation. *Obes. Rev.* 18 715–723. 10.1111/obr.12551 28489290

[B8] Brooks G. A. White P. (2018). Determination of metabolic and heart rate responses of rats to treadmill exercise. *J. Appl. Physiol. Respir. Environ. Exerc. Physiol.* 45 1009–1015. 10.1152/jappl.1978.45.6.1009 730582

[B9] Chait A. den Hartigh L. J. (2020). Adipose tissue distribution, inflammation and its metabolic consequences, including diabetes and cardiovascular disease. *Front. Cardiovasc. Med.* 7:22. 10.3389/fcvm.2020.00022 32158768 PMC7052117

[B10] Chang J. S. Kim T. H. Nguyen T. T. Park K. S. Kim N. Kong I. D. (2017). Circulating irisin levels as a predictive biomarker for sarcopenia: a cross-sectional community-based study. *Geriatr. Gerontol. Int.* 17 2266–2273. 10.1111/ggi.13030 28394089

[B11] Cheng L. Wang J. Dai H. Duan Y. An Y. Shi L. (2021). Brown and beige adipose tissue: a novel therapeutic strategy for obesity and type 2 diabetes mellitus. *Adipocyte* 10 48–65. 10.1080/21623945.2020.1870060 33403891 PMC7801117

[B12] Chusyd D. E. Wang D. Huffman D. M. Nagy T. R. (2016). Relationships between rodent white adipose fat pads and human white adipose fat depots. *Front. Nutr.* 3:10. 10.3389/fnut.2016.00010 27148535 PMC4835715

[B13] Costa L. R. de Castro C. A. Marine D. A. Fabrizzi F. Furino V. O. Malavazi I. (2021). High-Intensity interval training does not change vaspin and omentin and does not reduce visceral adipose tissue in obese rats. *Front. Physiol.* 12:564862. 10.3389/fphys.2021.564862 33716759 PMC7952996

[B14] Crujeiras A. B. Pardo M. Roca-Rivada A. Navas-Carretero S. Zulet M. A. Martínez J. A. (2014). Longitudinal variation of circulating irisin after an energy restriction-induced weight loss and following weight regain in obese men and women. *Am. J. Hum. Biol.* 26 198–207. 10.1002/ajhb.22493 24375850

[B15] De Macedo S. M. Lelis D. F. Mendes K. L. Fraga C. A. C. Brandi I. V. Feltenberger J. D. (2017). Effects of dietary macronutrient composition on FNDC5 and Irisin in mice skeletal muscle. *Metab. Syndr. Relat. Disord.* 15 161–169. 10.1089/met.2016.0109 28437200

[B16] Deveaud C. Beauvoit B. Salin B. Schaeffer J. Rigoulet M. (2004). Regional differences in oxidative capacity of rat white adipose tissue are linked to the mitochondrial content of mature adipocytes. *Mol. Cell. Biochem.* 267 157–166. 10.1023/b:mcbi.0000049374.52989.9b 15663197

[B17] Ebert T. Focke D. Petroff D. Wurst U. Richter J. Bachmann A. (2014). Serum levels of the myokine irisin in relation to metabolic and renal function. *Eur. J. Endocrinol.* 170 501–506. 24399249 10.1530/EJE-13-1053

[B18] Elkin L. A. Kay M. Higgins J. J. Wobbrock J. O. (2021). *An Aligned Rank Transform Procedure for Multifactor Contrast Tests. Proceedings of. Association for Computing Machinery*, Vol. 1. Available online at: http://arxiv.org/abs/2102.11824 10.1530/eje-13-1053 (accessed May 02, 2021). 24399249

[B19] Estadella D. Oyama L. M. Dâmaso A. R. Ribeiro E. B. Oller Do Nascimento C. M. (2004). Effect of palatable hyperlipidic diet on lipid metabolism of sedentary and exercised rats. *Nutrition* 20 218–224. 10.1016/j.nut.2003.10.008 14962690

[B20] Fagundo A. B. Jiménez-Murcia S. Giner-Bartolomé C. Agüera Z. Sauchelli S. Pardo M. (2016). Modulation of irisin and physical activity on executive functions in obesity and morbid obesity. *Sci. Rep.* 6:30820. 10.1038/srep30820 PMC4967861 27476477

[B21] Fatouros I. G. (2018). Is irisin the new player in exercise-induced adaptations or not? A 2017 update. *Clin. Chem. Lab. Med.* 56 525–548. 10.1515/cclm-2017-0674 29127759

[B22] Ferland D. J. GArver H. Contreras G. A. Fink G. D. Watts W. (2020). Chemerin contributes to in vivo adipogenesis in a location-specific manner. *PLoS One* 15:e0229251. 10.1371/journal.pone.0229251 32092101 PMC7039425

[B23] Frühbeck G. Fernández-Quintana B. Paniagua M. Hernández-Pardos A. W. Valentí V. Moncada R. (2020). FNDC4, a novel adipokine that reduces lipogenesis and promotes fat browning in human visceral adipocytes. *Metabolism* 108:154261. 10.1016/j.metabol.2020.154261 32407726

[B24] Fukushima Y. Kurose S. Shinno H. Cao Thi Thu H. Tamanoi A. Tsutsumi H. (2016). Relationships between serum irisin levels and metabolic parameters in Japanese patients with obesity. *Obes. Sci. Pract.* 2 203–209. 10.1002/osp4.43 27840690 PMC5089593

[B25] Gao F. Zheng K. I. Zhu P. W. Li Y. Y. Ma H. L. Li G. (2020). FNDC5 polymorphism influences the association between sarcopenia and liver fibrosis in adults with biopsy-proven nonalcoholic fatty liver disease. *Br. J. Nutr.* 1–12. 10.1017/s0007114520004559 33198849

[B26] Gong H. Han Y. W. Sun L. Zhang Y. Zhang E. Y. Li Y. (2016). The effects of energy intake of four different feeding patterns in rats. *Exp. Biol. Med.* 241 52–59. 10.1177/1535370215584890 25966980 PMC4935427

[B27] Grygiel-Górniak B. Puszczewicz M. (2017). A review on irisin, a new protagonist that mediates muscle-adipose-bone-neuron connectivity. *Eur. Rev. Med. Pharmacol. Sci.* 21 4687–4693. 29131244

[B28] Guilford B. L. Parson J. C. Grote C. W. Vick S. N. Ryals J. M. Wright D. E. (2017). Increased FNDC5 is associated with insulin resistance in high fat-fed mice. *Physiol. Rep.* 5:e13319. 10.14814/phy2.13319 28676551 PMC5506519

[B29] Hecksteden A. Wegmann M. Steffen A. Kraushaar J. Morsch A. Ruppenthal S. (2013). Irisin and exercise training in humans - Results from a randomized controlled training trial. *BMC Med.* 11:235. 10.1186/1741-7015-11-235 24191966 PMC4228275

[B30] Hew-Butler T. Landis-Piwowar K. Byrd G. Seimer M. Seigneurie N. Byrd B. (2015). Plasma irisin in runners and nonrunners: No favorable metabolic associations in humans. *Physiol. Rep.* 3:e12262. 10.14814/phy2.12262 25602017 PMC4387758

[B31] Huh J. Y. Dincer F. Mesfum E. Mantzoros C. S. (2014). Irisin stimulates muscle growth-related genes and regulates adipocyte differentiation and metabolism in humans. *Int. J. Obes.* 38 1538–1544. 10.1038/ijo.2014.42 24614098

[B32] Huh J. Y. Panagiotou G. Mougios V. Brinkoetter M. Vamvini M. T. Benjamin E. S. (2012). FNDC5 and irisin in humans: I. Predictors of circulating concentrations in serum and plasma and II. mRNA expression and circulating concentrations in response to weight loss and exercise. *Metabolism* 61 1725–1738. 10.1016/j.metabol.2012.09.002 23018146 PMC3614417

[B33] Kahn C. R. Wang G. Lee K. Y. (2019). Altered adipose tissue and adipocyte function in the pathogenesis of metabolic syndrome. *J. Clin. Invest.* 129 3990–4000. 10.1172/jci129187 31573548 PMC6763230

[B34] Kang Y. S. Kim J. C. Kim J. S. Kim S. H. (2019). Effects of swimming exercise on serum irisin and bone FNDC5 in rat models of high-fat diet-induced osteoporosis. *J. Sport Sci. Med.* 18 596–603. PMC6873128 31827343

[B35] Kazeminasab F. Marandi S. M. Ghaedi K. Safaeinejad Z. Esfarjani F. Nasr-Esfahani M. H. (2018). A comparative study on the effects of high-fat diet and endurance training on the PGC-1α-FNDC5/irisin pathway in obese and nonobese male C57BL/6 mice. *Appl. Physiol. Nutr. Metab.* 43 651–662. 10.1139/apnm-2017-0614 29365291

[B36] Khalafi M. Mohebbi H. Symonds M. E. Karimi P. Akbari A. Tabari E. (2020). The impact of moderate-intensity continuous or high-intensity interval training on adipogenesis and browning of subcutaneous adipose tissue in obese male rats. *Nutrients* 12:925. 10.3390/nu12040925 32230849 PMC7231004

[B37] Kim H. J. Lee H. J. So B. Son J. S. Yoon D. Song W. (2016). Effect of aerobic training and resistance training on circulating irisin level and their association with change of body composition in overweight/obese adults: a pilot study. *Physiol. Res.* 65 271–279. 10.33549/physiolres.932997 26447516

[B38] Kim H. Wrann C. D. Jedrychowski M. P. Sara V. Yukiko K. Nagano K. (2018). Irisin mediates effects on bone and fat via αV integrin receptors. *Cell* 175 1756–1768. 10.1016/j.cell.2018.10.025 30550785 PMC6298040

[B39] Kirat D. Hamada M. Moustafa A. Miyasho T. (2021). Irisin/FNDC5: a participant in camel metabolism. *Saudi J. Biol. Sci.* 28 693–706. 10.1016/j.sjbs.2020.10.061 33424357 PMC7783842

[B40] Kusminski C. M. Bickel P. E. Scherer P. E. (2016). Targeting adipose tissue in the treatment of obesity-associated diabetes. *Nat. Rev. Drug Discov.* 15 639–660. 10.1038/nrd.2016.75 27256476

[B41] Lee H. J. Lee J. O. Kim N. Kim J. K. Kim H. I. Lee Y. W. (2015). Irisin, a novel myokine, regulates glucose uptake in skeletal muscle cells via AMPK. *Mol. Endocrinol.* 29 873–881. 10.1210/me.2014-1353 25826445 PMC5414737

[B42] Liu S. Du F. Li X. Wang M. Duan R. Zhang J. (2017). Effects and underlying mechanisms of irisin on the proliferation and apoptosis of pancreatic β cells. *PLoS One* 12:e0175498. 10.1371/journal.pone.0175498 28394923 PMC5386279

[B43] Lu Y. Li G. (2020). Auricular acupuncture induces FNDC5/irisin and attenuates obese inflammation in mice. *Acupunct. Med.* 38 264–271. 32195595 10.1136/acupmed-2017-011405

[B44] Lu Y. Li H. Shen S.-W. Shen Z.-H. Xu M. Yang C.-J. (2016). Swimming exercise increases serum irisin level and reduces body fat mass in high-fat-diet fed Wistar rats. *Lipids Health Dis.* 15:93. 10.1186/s12944-016-0263-y PMC4866429 27177924

[B45] Luo Y. Qiao X. Ma Y. Deng H. Xu C. C. Xu L. (2020). Disordered metabolism in mice lacking irisin. *Sci. Rep.* 10:17368. 10.1038/s41598-020-74588-7 PMC7567109 33060792

[B46] Matthews D. R. Hosker J. P. Rudenski A. S. Naylor B. A. Treacher D. F. Turner R. C. (1985). Homeostasis model assessment: insulin resistance and β-cell function from fasting plasma glucose and insulin concentrations in man. *Diabetologia* 28 412–419. 10.1007/bf00280883 3899825

[B47] Moreno-Navarrete J. M. Ortega F. Serrano M. Guerra E. Pardo G. Tinahones F. (2013). Irisin is expressed and produced by human muscle and adipose tissue in association with obesity and insulin resistance. *J. Clin. Endocrinol. Metab.* 98 E769–E778. 23436919 10.1210/jc.2012-2749

[B48] Mukaka M. M. (2012). Statistics corner: a guide to appropriate use of correlation coefficient in medical research. *Malawi Med. J.* 24 69–71. 23638278 PMC3576830

[B49] Norheim F. Langleite T. M. Hjorth M. Holen T. Kielland A. Stadheim H. K. (2014). The effects of acute and chronic exercise on PGC-1a, irisin and browning of subcutaneous adipose tissue in humans. *FEBS J.* 281 739–749. 10.1111/febs.12619 24237962

[B50] Oishi J. C. Castro C. A. Silva K. A. Fabricio V. Cárnio E. C. Phillips S. A. (2018). Endothelial dysfunction and inflammation precedes elevations in blood pressure induced by a high-fat diet. *Arq. Bras. Cardiol.* 110 558–567. 30226915 10.5935/abc.20180086 PMC6023639

[B51] Pardo M. Crujeiras A. B. Amil M. Aguera Z. Jiménez-Murcia S. Baños R. (2014). Association of irisin with fat mass, resting energy expenditure, and daily activity in conditions of extreme body mass index. *Int. J. Endocrinol.* 2014:857270. 10.1155/2014/857270 PMC4016898 24864142

[B52] Park K. H. Zaichenko L. Brinkoetter M. Thakkar B. Sahin-Efe A. Joung K. E. (2013). Circulating irisin in relation to insulin resistance and the metabolic syndrome. *J. Clin. Endocrinol. Metab.* 98 4899–4907. 10.1210/jc.2013-2373 24057291 PMC3849667

[B53] Perakakis N. Triantafyllou G. A. Fernández-Real J. M. Huh J. Y. Park K. H. Seufert J. (2017). Physiology and role of irisin in glucose homeostasis. *Nat. Rev. Endocrinol.* 13 324–337. 10.1038/nrendo.2016.221 28211512 PMC5878942

[B54] Quiñones M. Folgueira C. Sánchez-Rebordelo E. Al-Massadi O. (2015). Circulating Irisin levels are not regulated by nutritional status, obesity, or leptin levels in rodents. *Mediators Inflamm.* 2015:620919. 10.1155/2015/620919 PMC4629051 26568663

[B55] Roca-Rivada A. Castelao C. Senin L. L. Landrove M. O. Baltar J. Crujeiras A. B. (2013). FNDC5/Irisin is not only a myokine but also an adipokine. *PLoS One* 8:e60563. 10.1371/journal.pone.0060563 23593248 PMC3623960

[B56] Rodríguez A. Becerril S. Ezquerro S. Méndez-Giménez L. Frühbeck G. (2017). Crosstalk between adipokines and myokines in fat browning. *Acta Physiol.* 219 362–381. 10.1111/apha.12686 27040995

[B57] Rodríguez A. Becerril S. Méndez-Giménez L. Ramírez B. Sáinz N. Catalán V. (2015). Leptin administration activates irisin-induced myogenesis via nitric oxide-dependent mechanisms, but reduces its effect on subcutaneous fat browning in mice. *Int. J. Obes.* 39 397–407. 10.1038/ijo.2014.166 25199621

[B58] Romieu I. Dossus L. Barquera S. Blottière H. M. Franks P. W. Gunter M. (2017). Energy balance and obesity: what are the main drivers? *Cancer Causes Control* 28 247–258. 10.1007/s10552-017-0869-z 28210884 PMC5325830

[B59] Roza N. A. V. Possignolo L. F. Palanch A. C. Gontijo A. R. (2016). Effect of long-term high-fat diet intake on peripheral insulin sensibility, blood pressure, and renal function in female rats. *Food Nutr.* 60:28536. 10.3402/fnr.v60.28536 26880072 PMC4754019

[B60] Sahin-Efe A. Upadhyay J. Ko B. J. Dincer F. Park K. H. Migdal A. (2018). Irisin and leptin concentrations in relation to obesity, and developing type 2 diabetes: a cross sectional and a prospective case-control study nested in the Normative Aging Study. *Metabolism* 79 24–32. 10.1016/j.metabol.2017.10.011 29108900

[B61] Schoettl T. Fischer I. P. Ussar S. (2018). Heterogeneity of adipose tissue in development and metabolic function. *J. Exp. Biol.* 221 ((Pt Suppl. 1)):17. 10.1242/jeb.162958 29514879

[B62] Souza R. W. A. Alves C. R. R. Medeiros A. Rolim N. Silva G. J. J. Moreira J. B. N. (2018). Differential regulation of cysteine oxidative post-translational modifications in high and low aerobic capacity. *Sci. Rep.* 8:17772. 10.1038/s41598-018-35728-2 PMC6289973 30538258

[B63] Stengel A. Hofmann T. Goebel-Stengel M. Elbelt U. Kobelt P. Klapp B. F. (2013). Circulating levels of irisin in patients with anorexia nervosa and different stages of obesity-Correlation with body mass index. *Peptides* 39 125–130. 10.1016/j.peptides.2012.11.014 23219488

[B64] Tang L. Tong Y. Zhang F. Chen G. Zhang Y. C. Jobin J. (2019). The association of circulating irisin with metabolic risk factors in Chinese adults: a cross-sectional community-based study. *BMC Endocr. Disord.* 19:147. 10.1186/s12902-019-0479-8 31881940 PMC6935078

[B65] Varela-Rodríguez B. M. Pena-Bello L. Juiz-Valiña P. Vidal-Bretal B. Cordido F. Sangiao-Alvarellos S. (2016). FNDC5 expression and circulating irisin levels are modified by diet and hormonal conditions in hypothalamus, adipose tissue and muscle. *Sci. Rep.* 6:29898. 10.1038/srep29898 PMC4949437 27432282

[B66] Verboven K. Wouters K. Gaens K. Hansen D. Bijnen M. Wetzels S. (2018). Abdominal subcutaneous and visceral adipocyte size, lipolysis and inflammation relate to insulin resistance in male obese humans. *Sci. Rep.* 8 1–8. 10.1038/s41598-018-22962-x 29549282 PMC5856747

[B67] Verheggen R. J. H. M. Maessen M. F. H. Green D. J. Hermus A. R. M. M. Hopman M. T. E. Thijssen D. H. T. (2016). A systematic review and meta-analysis on the effects of exercise training versus hypocaloric diet: distinct effects on body weight and visceral adipose tissue. *Obes. Rev.* 17 664–690. 10.1111/obr.12406 27213481

[B68] Wajchenberg B. L. (2000). Subcutaneous and visceral adipose tissue: their relation to the metabolic syndrome. *Endocr. Rev.* 21 697–738. 10.1210/edrv.21.6.0415 11133069

[B69] Willoughby D. Hewlings S. Kalman D. (2018). Body composition changes in weight loss: strategies and supplementation for maintaining lean body mass, a brief review. *Nutrients* 10:1876. 10.3390/nu10121876 30513859 PMC6315740

[B70] Wronska A. Kmiec Z. (2012). Structural and biochemical characteristics of various white adipose tissue depots. *Acta Physiol.* 205 194–208. 10.1111/j.1748-1716.2012.02409.x 22226221

[B71] Yang Z. Chen X. Chen Y. Zhao Q. (2015). Decreased Irisin secretion contributes to muscle insulin resistance in high-fat diet mice. *Int. J. Clin. Exp. Pathol.* 8 6490–6497. 26261526 PMC4525860

